# Considerations about ethical and legal aspects at the end of life during the COVID-19 pandemic

**DOI:** 10.6061/clinics/2021/e2821

**Published:** 2021-03-19

**Authors:** Ivan Dieb Miziara, Carmen Silvia Molleis Galego Miziara

**Affiliations:** I Medicina Legal, Etica Medica e Medicina Social e do Trabalho, Faculdade de Medicina FMUSP, Universidade de Sao Paulo, Sao Paulo, SP, BR.; II Laboratorio de Investigacao Medica 40, Instituto Oscar Freire, Hospital das Clinicas HCFMUSP, Faculdade de Medicina, Universidade de Sao Paulo, Sao Paulo, SP, BR.

## INTRODUCTION

The Sars-CoV-2 pandemic which has plagued the planet and in particular, Brazil, has killed more than 200,000 people so far. It has placed the discussion about some procedures to be adopted on the agenda, such as those for when a patient is approaching the end of their life, bedridden in an ICU bed, isolated in an infirmary, and is on their deathbed but remains alive with their anxieties, uncertainties, and feelings of abandonment.

During the first week of January 2021, the Jornal da USP (1) published a report in which students at the boarding school of the Faculty of Medicine of the University of São Paulo, engaged as volunteers to combat COVID-19, stimulated a debate on the practices of palliative care in the undergraduate curriculum under the claim that ideally, “such care should start when a person is diagnosed with a disease that compromises their life.”

Both issues (the right to a dignified death and the implementation of systematic teaching of palliative care in medical education) generate some pertinent reflections within the tragedy that we are all experiencing. The first of these reflections is about how to deal with the end of a patient’s life—bringing up primordial issues that are often covered up, such as orthothanasia, dysthanasia, and euthanasia—both ethically and legally.

The second issue that requires reflection concerns the point raised by undergraduate medical students about the opportunity for initial palliative care as well as the inclusion of this discipline in medical curriculum.

Regarding the first topic, some observations must be made a *priori*. William Frankena (2), a bioethicist at the University of Michigan, gives us the example of the philosopher Socrates (in one of Plato’s dialogs - “Críton”) when he was faced with the opportunity to escape from prison with the help of friends and save himself and his family. “We must not allow ourselves to be dominated by emotions, but to follow the best logical and moral reasoning. We must not get carried away by what most people believe is right, because they may be mistaken; we must reason for ourselves. Finally, we must not do anything that is morally wrong.” And, by obvious deduction, we can say, “We must do what is morally right.”

Starting from the Socratic premise that “we must not do the morally wrong,” in relation to the ethical issues about the need to have a dignified death, Levine (3) points out that “for decades, we have been trying to put this ideal into practice. Undoubtedly, palliative care currently makes a difference for some people, even though death is still accompanied by unwanted and ineffective interventions, excruciating pain and suffering, and loss of personal dignity and autonomy.”

The author also recalls that lawyers have recommended that people prepare their so-called “vital wills” or “advance will guidelines” as a precaution against useless and potentially painful treatments. However, he warns that there are criticisms about the “authenticity” of these documents (since the person who signed it is no longer the same person at the present moment of a terminal illness), not to mention the legal issue surrounding this whole problem. However, this is a long and separate discussion that will not be our topic for now. It is enough to say that the living will or advance will guidelines are now fully accepted and recommended by the Federal Council of Medicine.

From this point of view, it is our impression that the intersection of ethics, bioethics, and legal issues is not new. As Hall and King (4) state, “in the 1970s and 1980s legal disputes over the right to death dominated discussions, culminating in the 1990s with the decisions of the American Supreme Court in the *Cruzan* (forced feeding) and *Glucksberg* (assisted suicide) cases.” It is precisely the legal issue, in our opinion, that should require more accurate attention from doctors: what is morally correct is not always legal, or protected by law.

This article aims to present some ethical and medico-legal aspects that involve the end of life and to discuss the pertinence of expanding the teaching of palliative care in medical education, as well as to discuss the ideal opportunity to start this practice when the patient faces a real threat to life.

## ETHICAL, MEDICAL, AND LEGAL ASPECTS

Several actions are possible in the face of a terminal illness. Each of them has its pros and cons, moral justifications, and legal objections. However, all of them are susceptible to some form of criticism and potentially capable of generating conflicts between the agents involved (health professionals, the patient’s family, and the patient himself or herself). To make matters worse, even within our inner selves, conflicts can arise and often arouse doubts.

Imagine a situation like the one proposed by Hester (5), when we are thinking of acting for the benefit of a patient: suppose that you consider yourself a good person, raised with good principles of conduct and values, and that you are an individual who respects the laws in our country. Suppose that you are caring for a terminal patient with metastatic cancer, in which all available forms of therapy have been tried, including the last possible chemotherapy session. This patient is suffering from excruciating pain that makes their life a burden. In these conditions, moved by your good principles and a spirit of compassion, after obtaining consent from them, you decide to give a dose of lethal injection, with the sole purpose of shortening the suffering of the individual. This action is called active consent euthanasia (6).

However, it may be that your patient is in a state of unconsciousness or semi-consciousness, which does not allow them to give proper consent and still, you decide to give them the same lethal injection. In this case, the action is called active non-consenting euthanasia (6).

Now imagine another possible situation: your patient, who has an irreversible pulmonary condition as a result of Sars-CoV-2 infection, is under mechanical ventilation, and goes into a coma with acute renal failure and severe hemodynamic instability, characterizing a terminal condition. And, just for the sake of illustration, at that moment you decide not to take any additional therapeutic measures, knowing that there is nothing more to do for the patient in terms of prognosis. Some authors refer to this act as passive euthanasia (6).

On the other hand, one can adopt another type of conduct. Due to pressure from family members, or by the conviction of what your “doctor’s duty” is, you can subject the patient to some form of renal dialysis, you can increase the number of vasoactive drugs, and in this case, this attitude of maintaining life at any cost, prolonging the patient’s suffering with useless measures, is called dysthanasia (6).

Still, suppose that same patient develops septicemia and an apperceptive and non-responsive coma, without any electrical and metabolic brain activity, and you decide not to adopt any further therapeutic activity, letting the disease run its inexorable course, maintaining only clinical support and palliative care to avoid further suffering to the patient. This act is called orthothanasia (6).

From a moral point of view, it seems to us that both consenting to active euthanasia (although it is illegal) and orthothanasia are fully acceptable in the situations described above. Consented active euthanasia is a way of respecting the patient’s autonomy (despite all the contrary arguments that may exist, which we respect, and its illegality). Orthothanasia accompanied by appropriate palliative care, on the other hand, is a full expression of the Hippocratic principle of not causing harm to the patient.

Absolutely unacceptable (from a moral point of view) are active non-consenting euthanasia (also illegal in our country) and dysthanasia. We must always remember that the doctor’s obligation is to take care of the patient. The cure (or not) often depends on factors outside the doctor’s will: the patient, the stage of advancing medical knowledge about diseases, therapeutic forms, etc.

However, it is necessary to emphasize that, from a strictly legal point of view, the Brazilian Penal Code, in article 121 - “killing someone” - punishes the crime of death with a penalty of six to twenty years in prison. Even in cases of euthanasia—as discussed in the first paragraph of this article—which says: “If the agent commits the crime impelled by reason of relevant social or moral value,” the crime persists, even though the sentence is reduced from one-third to one-sixth, at the discretion of the judge.

In summary, in Brazil, euthanasia is characterized as homicide, although in a euphemistic way, it can be cataloged as “pious homicide.” As Genival Veloso França (7) rightly states, “our Code does not accept death out of compassion as an exclusive form of crime: it only gives the Judge the power to reduce the sentence.”

In turn, Lippmann (8), establishes that euthanasia is defined as “the realization of death at the request of the patient and, in Brazil, it is prohibited both by law and by medical ethics.” Dysthanasia, on the other hand, is “postponing death, despite the patient’s suffering,” and orthothanasia “is the possibility of a dignified death, letting nature take its course, according to the patient’s wishes and knowledge family members.”

We must remember that orthothanasia, according to Resolution 1,805/2006 of the Federal Council of Medicine, does not aim to cause the patient’s death. It is not about fighting death with the use of excessive and disproportionate technology, nor is it about shortening life through external action. It is characterized, therefore, by the use of palliative care procedures, to bring comfort, relief from pain, depression, and other symptoms responsible for the patient’s suffering in this final stretch of life. The first objective, Lippmann (8), “is to offer maximum comfort to the patient, without any intention of causing death.”

However, this position is not a challenge. França (7) argues that it is necessary to “distinguish what ordinary and extraordinary procedures mean” (or, as Lippmann (8) states, “excessive and disproportionate technology”).

The nature of what is ordinary and what is extraordinary has remained debatable since the 1960s. Emanuel (9) states that “ordinary means all drugs, treatments, and operations that offer a reasonable hope of benefit to the patient and that can be obtained and used without excessive cost, pain, or other inconveniences; while extraordinary means all medications, treatments, and operations that cannot be obtained without excessive cost, pain, or other type of inconvenience, and that, if used, do not bring a reasonable hope of benefit to the patient.”

However, this is not necessarily simple. Berlinger et al. (10), for example, argue that “medications and procedures that are routinely used in hospitals (such as blood transfusions and state-of-the-art antibiotics) do not mean that they should always be used. When the patient’s condition deteriorates, ‘routine’ procedures and medications can have their benefits reduced or ceased and cause discomfort and suffering.”

In our opinion, we believe that even intuitively, we all know how to differentiate, in medical practice, what is usual, common, and which will bring greater comfort to the patient, from what is futile, out of time, and which will bring greater suffering and pain. In these cases, we firmly believe that the physician’s autonomy must be preserved, and the help of the palliative care team is essential. If the same terminal cancer patient, as previously exemplified, requires a tracheostomy and family members refuse (considering the procedure unnecessary and causing prolonged suffering), the doctor may disagree with the family members, since this procedure, in addition to being common and necessary, will bring greater comfort to the patient. The intermediation of the palliative care team at this time is crucial.

 In contrast, for patients with irreversible pulmonary symptoms due to Sars-CoV-2 who require dialysis sessions due to the overlapping of renal insufficiency, this procedure should be considered extraordinary as shown above, and the issue should be discussed with family members, the palliative care team, and evaluated from the perspective of the principle of justice, considering that such a procedure could cause more suffering to the patient, besides being able to generate losses to other patients who could benefit from dialysis devices, which may have reduced availability in medical facilities across the country.

## TEACHING PALLIATIVE CARE

According to the World Health Organization (11), “palliative care is an approach that improves the quality of life of patients (adults and children) and family members when they face problems inherent to a potentially deadly disease.” As Pegoraro and Paganini (12) state, “this approach is related to caring for life, regardless of its duration. It intends to rescue the dignity and will of the terminal patient.”

In a previous publication (6), we made it clear that these procedures go hand in hand with orthothanasia and it is a moral duty of the medical professional - whose primary objective, as we said, is to care for the patient, to welcome them, and minimize their suffering. The opportunity to start this type of care, in our understanding, must also be taken as early as possible in the face of diseases that appear as direct threats to life.

We believe that with the advancement of technological means of supporting life and the possibility of keeping a living organism indefinitely with all the ethical consequences that this entails, it is increasingly important to discuss, debate, and teach undergraduate students the methods and procedures of palliative care. We see such procedures as complex and requiring specialized action; however, these can and should still be taught to those who graduate in medicine, even if they do not become specialists in this or related areas.

As stated by Fonseca and Geovanini (13), “Talking about death in graduation involves addressing training in skills such as communication, teamwork, and family support, in addition to the control of signs and symptoms, so that you can offer quality and end-of-life care and minimize the suffering of those facing the terminal phase of the disease. The inclusion of palliative care in undergraduate medical education is an option to be discussed in the current curricula so that one can stimulate the specialized technical capacity in this area of knowledge and spread the care techniques for any medical specialty.”

In order to have an idea of undergraduate students’ lack of knowledge about topics that involve the terminality of life, in a recent study, Pereira et al. (14) demonstrated that only 43% of sixth-year students of a medical school in Goiás knew how to define what was dysthanasia and 74% of these students reported a deficit in addressing the topic of palliative care in medical education. Despite being an isolated example, and obviously with some interpretation bias, we believe that there is a global representation in these papers regarding the lack of knowledge about end-of-life issues as well as palliative care among undergraduate students of medicine.

Our population is aging, we have an increased prevalence of chronic diseases, and seems to us that is important in bringing competence, skills, and training of future health professionals in palliative medicine. Palliative care involves a multi-professional approach that is patient-centered and requires specific clinical skills. There is a huge list of skills to be developed among medical students. They have to learn the best form of communication with the patient and the family; they have to develop many skills in the quality of end-of-life care, and learn how to control pain and other symptoms. The need to improve education in palliative care has been well documented worldwide.

## FINAL CONSIDERATIONS

The COVID-19 epidemic that hit our country violently raised questions about various aspects related to end-of-life issues. Ethical, and legal questions that uncover the lack of knowledge of undergraduate medical students regarding palliative care techniques were raised.

We tried to examine the parameters of legal conduct within our legal system, and express our point of view on the moral justification of certain acts, without intending to exhaust the subject that is and will be increasingly the subject of in-depth debates, with the sole purpose of improving medical practice.

Regarding the teaching of palliative care in medical schools, it is necessary, first, to assess the degree of knowledge of the students on the topic, to establish clear learning goals, and to choose appropriate teaching and learning methods. This is a challenge for the future that none of us can ignore.

## AUTHOR CONTRIBUTIONS

Miziara ID wrote the manuscript, with suggestions from Miziara CSMG, who also reviewed the content and grammar.
